# Exploring the Causes of Missed Appointments at Ibra Hospital in the Sultanate of Oman: A Cross-Sectional Study

**DOI:** 10.7759/cureus.96541

**Published:** 2025-11-11

**Authors:** Faisal Al Sawafi, Musleh M Al Musalhi, Kamel Mubarak Al Sheheimi, Ahmed Said Al Falahi, Sheikha Abdullah Al Sudairi

**Affiliations:** 1 Critical Care, Ibra Hospital, Ibra, OMN; 2 Hematopathology, Ibra Hospital, Ibra, OMN; 3 Biostatistics, Ibra Hospital, Ibra, OMN; 4 Nursing, Ibra Hospital, Ibra, OMN

**Keywords:** appointment, defaulter, hospital visit, observational cross-sectional study, outpatient visits

## Abstract

Objectives

Understanding the causes behind missed outpatient appointments is essential for implementing targeted interventions to improve patient attendance and healthcare delivery. This study aimed to explore the reasons for missed appointments at Ibra Hospital, Sultanate of Oman, from the perspectives of both patients and healthcare workers.

Methods

A cross-sectional study was conducted involving 420 adult patients who missed scheduled OPD appointments across various clinics, including general surgery, ENT, dermatology, and others, at Ibra Hospital between January and June 2025. Patients or their guardians were contacted via telephone using a structured questionnaire adapted from prior research. Additionally, healthcare workers were surveyed to gather their perspectives on the causes of missed appointments. Descriptive statistics were used to summarize department-wise defaulters and reasons for nonattendance.

Results

General surgery (17.03%), ENT (16.79%), and dermatology (14.32%) clinics reported the highest number of missed appointments. The most common reasons cited for missed appointments included forgetting the appointment (12.09%), lack of leave from work (9.13%), transportation problems (6.41%), and rescheduling (6.91%). Notably, 29.87% of participants provided no reason or did not respond; further inquiry revealed explanations such as clinical improvement (17.9%) and bypassing the appointment system by attending as walk-ins (16.4%). Healthcare staff identified transportation difficulties and communication gaps as major contributing factors.

Conclusions

Missed appointments at Ibra Hospital are influenced by a combination of forgetfulness, work constraints, logistical challenges, and system-related issues. Tailored interventions could help reduce appointment nonattendance, thereby improving hospital efficiency and continuity of patient care. Further multi-center studies are recommended to validate and generalize these findings across Oman.

## Introduction

Missed appointments in outpatient departments pose a multifaceted challenge to hospitals, impacting operational efficiency, financial stability, and patient health outcomes. Addressing this issue requires a comprehensive approach that includes enhancing patient engagement, implementing effective reminder systems, and providing support to overcome socioeconomic barriers. By addressing the root causes of missed appointments, hospitals can improve their overall efficiency and the quality of care they deliver to patients [1].

Missed appointments, often referred to as “no-shows,” in hospital outpatient departments represent a significant challenge to the healthcare system [2,3]. They affect various aspects of healthcare delivery, from operational efficiency and financial stability to patient health outcomes. Missed appointments disrupt scheduling and the smooth operation of outpatient departments. Healthcare providers allocate specific time slots for each patient, and when a patient does not show up, that time is wasted. This inefficiency leads to several issues. These missed appointments waste the time of medical staff, including doctors, nurses, and administrative personnel, time that could otherwise be utilized for other patients. Rescheduling missed appointments results in longer waiting periods for other patients who need timely care. Frequent missed appointments by certain patients contribute to a disorganized workflow, making it difficult for staff to manage their time and resources effectively [4,5].

Missed appointments also have substantial financial repercussions for hospitals. Each missed appointment leads to a direct loss of revenue. For hospitals that rely on patient visits to sustain operations, these losses can accumulate significantly on a weekly, monthly, and annual basis. Hospitals may also incur additional administrative costs in managing and rescheduling no-shows. Some specialties may overbook appointments to compensate for expected missed visits, which can overburden staff and resources if all patients attend. The most critical impact of missed appointments is on patient health. Missing scheduled appointments can lead to delays in diagnosis and treatment, worsening medical conditions and resulting in poorer health outcomes (e.g., renal failure, diabetes mellitus, coronary artery disease). Regular follow-ups are essential for managing chronic conditions and monitoring treatment effectiveness. Missed appointments disrupt the continuity of care, making it difficult for healthcare providers to maintain comprehensive and consistent patient management. Patients who miss outpatient appointments may eventually require more urgent care, leading to an increase in emergency department visits. This not only places additional strain on emergency services but also results in higher healthcare costs for both patients and hospitals [6-8].

Several psychosocial and behavioral factors contribute to missed appointments. Patients from lower socioeconomic backgrounds may face challenges such as a lack of transportation, inflexible work schedules, or the unavailability of an accompanying attendee, leading to higher rates of missed appointments. Patients with limited health literacy may not fully understand the importance of keeping their appointments or the potential implications of missing them. Medical conditions such as depression and anxiety can also affect a patient’s ability to adhere to scheduled appointments. Hospitals can implement several strategies to reduce the incidence of missed appointments. Automated reminders through phone calls, text messages, WhatsApp, or email can significantly reduce no-show rates by reminding patients of their upcoming visits [9,10].

Educating patients about the importance of their appointments and involving them in their care plans can improve attendance at respective clinics. For some patients, flexible scheduling options, such as extended hours or telehealth consultations, can help minimize missed appointments. Providing information about, or assistance with, transportation options can also support patients who face logistical barriers [11].

This study aims to identify the reasons for missed hospital appointments from the perspectives of patients across various outpatient clinics. The main objectives were to compile a list of patients who missed appointments during the specified period, to contact eligible patients via telephone to determine their reasons for missing scheduled appointments, and to capture the perspectives of healthcare workers through a structured questionnaire.

## Materials and methods

This study was approved by the Directorate General of Health Services, North Sharqiya Governorate (DGHS-NSH), Sultanate of Oman (proposal ID: MoH/CSR/24/28822). It was conducted at Ibra Hospital, a secondary care hospital in Wilayat Ibra, North Sharqiya Governorate, Sultanate of Oman. Adult patients who missed OPD appointments were considered eligible. These patients were from various outpatient departments, including medicine, general surgery, orthopedics, ENT, ophthalmology, dermatology, and obstetrics.

We used the questionnaire from the article by Alawadhi A et al. as a reference [12]. Based on the questions used in that study, we prepared 17 questions. These were circulated as a Google Form to the heads of various clinical departments, hospital administrators, medical records staff, nursing in-charges, and doctors working in different departments. The face validity of the questions was established by circulating the initial set among experts in the field (nursing supervisors and medical records personnel). The content validity was established by circulating the revised set among hospital administrators, patient relations officers, and doctors. The questionnaire was finalized based on the responses and feedback from participants who completed the pilot version. The final questionnaire is presented in Table 1.

**Table 1:** List of questions included in the participant questionnaire.

No.	Question
1	Did you forget the appointment?
2	Were you/the patient sick on the day of the appointment?
3	Was there a transportation problem?
4	Did you reschedule for another date?
5	Were you unable to get leave from work?
6	Had you already attended the appointment or made one at another hospital?
7	Was there no one available to accompany you?
8	Did you have an exam or interview on that day?
9	Were you out of station or occupied with work commitments?
10	Did you receive an incomplete appointment message (e.g., missing name or time)?
11	Did you not receive an appointment message, or had your phone number changed?
12	Did you not attend because you could not understand the message, as it was in English?
13	Was there a family or social emergency (e.g., a death or relative being ill)?
14	Did you not receive any communication about appointment confirmation?
15	Did you receive any reminder for the appointment?
16	Were you sleeping and did not wake up in time?
17	Did you have other family commitments?
18	Was the appointment time not convenient?
19	Was there no specific reason for missing the appointment?
20	Did the hospital cancel the appointment?
21	Did you change the appointment through a relative or someone known in the hospital (e.g., hospital employee, administration, or doctor)?
22	Others / Miscellaneous.

Patients aged 18 years and above registered with the aforementioned clinics who missed scheduled appointments were contacted directly over the telephone. For patients below 18 years of age (pediatrics), their registered parents’ telephone numbers were contacted. Mentally challenged patients were excluded from the analysis due to the unique challenges they pose for transportation. Data were collected by the primary and co-investigators.

Sample size

As of January 2025, North Sharqiya Governorate had a population of approximately 304,232. For a 95% CI and a 5% margin of error, a sample size of 384 was required. Considering potential dropouts and incomplete responses, a final sample size of 400 was selected for this study.

Descriptive analyses were conducted for patient-reported reasons overall and by clinic. Staff-reported perceptions were also summarized. No regression modeling was planned. The responses and relevant data were entered into a Microsoft Excel sheet for analysis, and the statistical parameters used were frequencies and percentages. Independent predictors included age category, sex, appointment status (missed or attended), appointment day, appointment month, appointment year, and marital status.

## Results

**Figure 1:**
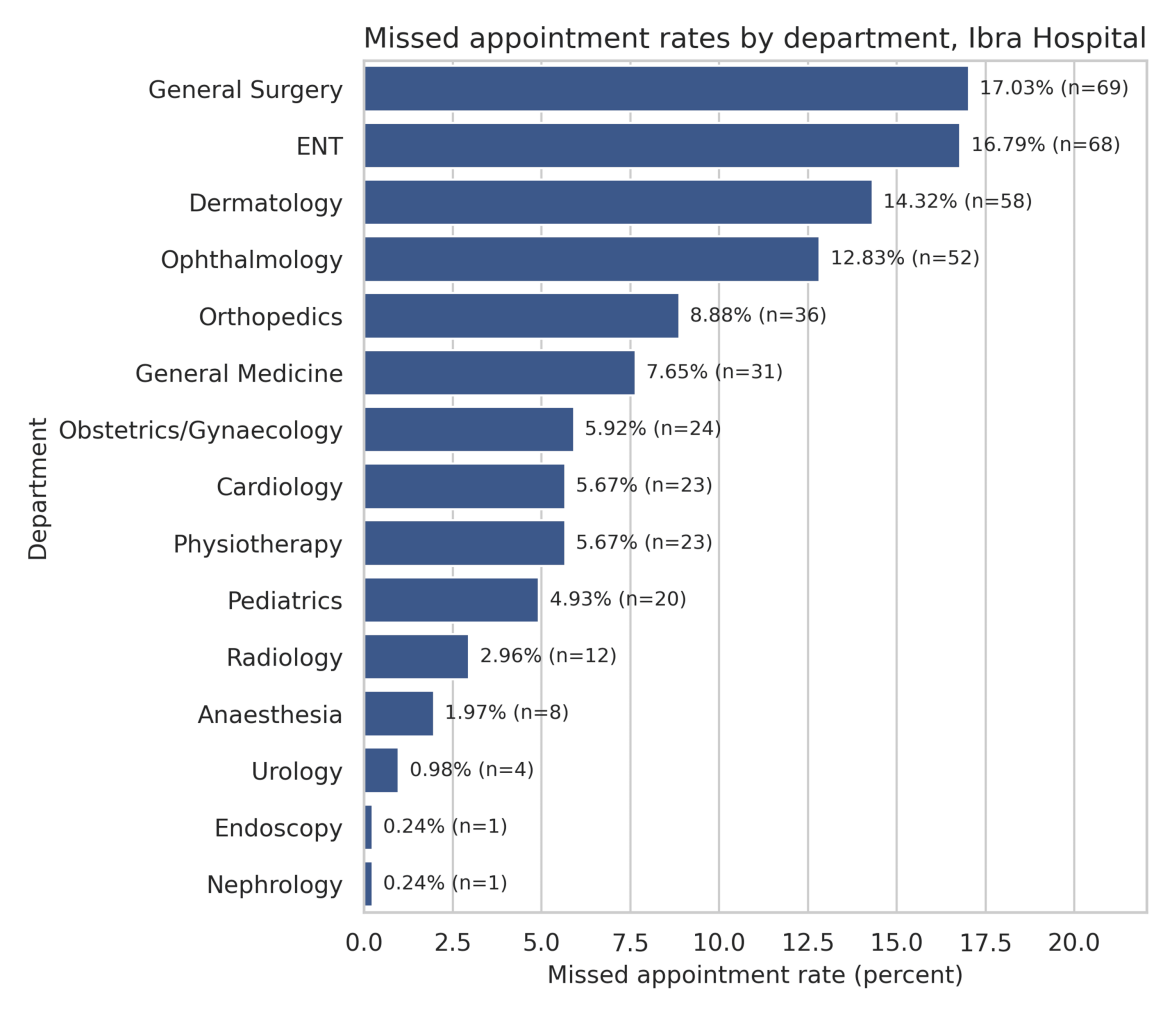
Department-wise summary of defaulters, expressed in numbers and percentages. Image generated using Julius AI (Version 2.1; Julius AI, Inc., https://julius.ai)

**Figure 2:**
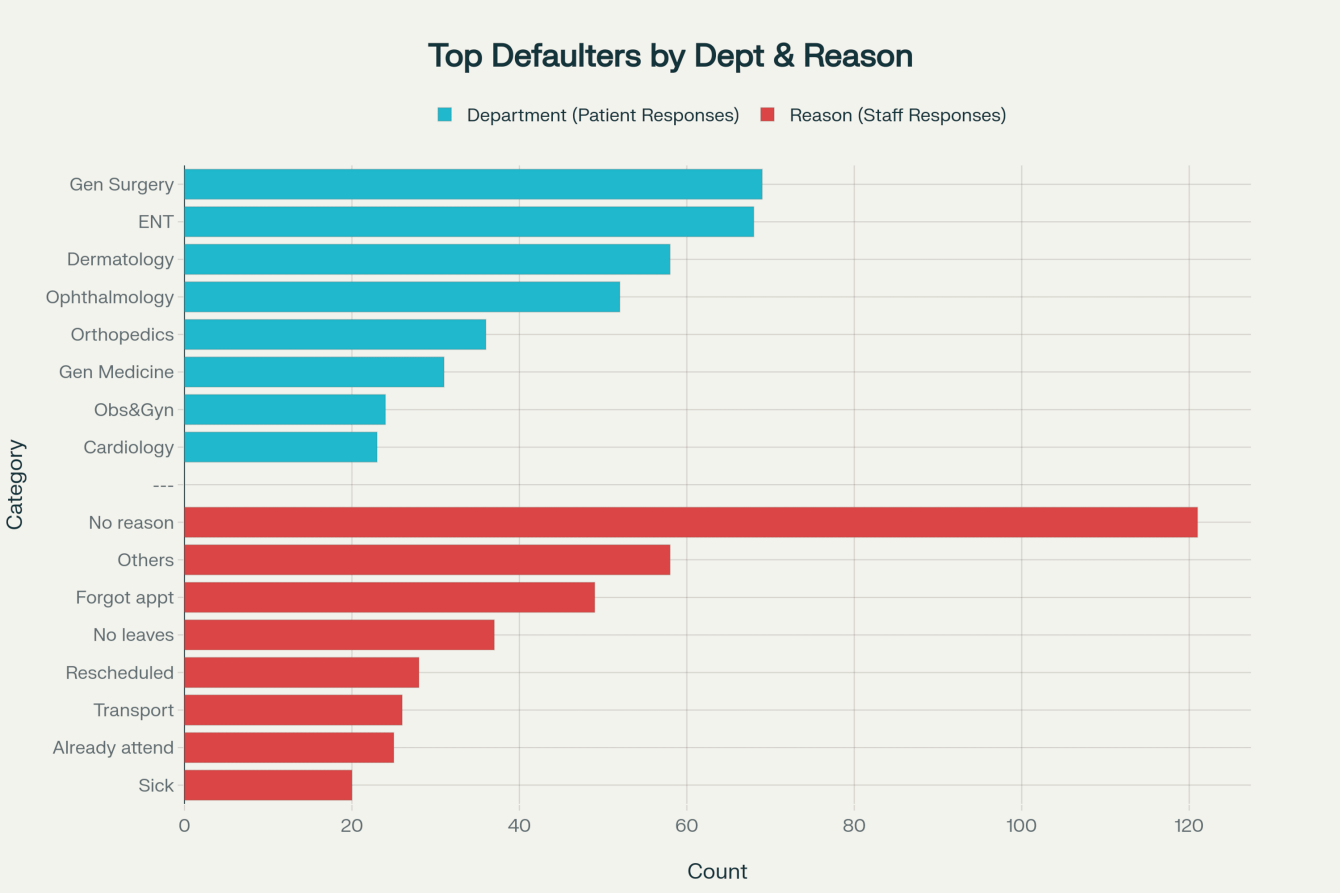
Comparative bar chart of patient and staff responses. Image generated using the Perplexity AI tool (https://www.perplexity.ai).

The defaulters’ list was retrieved from January to January 2025, until a total exceeding 400 cases was achieved. A total of 420 defaulters were contacted by various co-authors, and the responses were entered into an Excel sheet. Table 2 summarizes the department-wise distribution of defaulters. These patients represented various clinical departments, providing a comprehensive overview of missed appointment patterns across specialties. The highest number of defaulters was recorded in the Department of General Surgery (n = 69), followed by ENT (n = 68) and Dermatology (n = 58) (Figure 1). Figure 2 illustrates a comparison between patient and staff responses. The various reasons for being a defaulter for the scheduled appointment are summarized in Table 3.

**Table 2:** Department-wise summary of defaulters.

Department	Total Defaulters (n = 420)	Percentage (%)
Anaesthesia	8	1.97
Cardiology	23	5.67
Dermatology	58	14.32
Endoscopy	1	0.24
ENT	68	16.79
General Medicine	31	7.65
General Surgery	69	17.03
Nephrology	1	0.24
Obstetrics and Gynaecology	24	5.92
Ophthalmology	52	12.83
Orthopaedics	36	8.88
Paediatrics	20	4.93
Physiotherapy	23	5.67
Radiology	12	2.96
Urology	4	0.98

**Table 3:** Summary of reasons for defaulting.

Reason	Defaulters (n = 420)	Percentage (%)
Forgot appointment	49	12.09
Sick	20	4.93
Transportation problem	26	6.41
Rescheduled	28	6.91
No leave from work	37	9.13
Already attended / made another appointment	25	6.17
Exam / interview	13	3.2
Family emergency	7	1.72
No message received	20	4.93
Could not understand message	4	0.98
No reminder received	2	0.49
Family commitment	8	1.97
Inconvenient timing	6	1.48
No reason / no reply	121	29.87
Changed appointment	6	1.48
Others	58	14.32

As evident from the table, the category “no reason or reply” accounted for the highest number of defaulters (n = 121). Since these respondents had not provided a specific reason, they were contacted again to clarify their responses, which were possibly not included in the predefined list of options. Of the 121 defaulters, 67 (56.77%) provided additional explanations, as shown in Table 3. Among these, 12 defaulters mentioned that they felt clinically improved and, therefore, did not find it necessary to attend the appointment. Another 11 stated that they had already visited the hospital by bypassing the appointment system.

Other notable reasons included family emergencies, exams or interviews, family commitments, inconvenient appointment timings, and failure to receive messages or reminders. A small minority cited misunderstanding of instructions or dissatisfaction with previous treatment. Overall, these findings highlight the multifactorial and sometimes nuanced reasons contributing to missed attendance in outpatient settings.

## Discussion

One of the major challenges facing healthcare systems worldwide is missed appointments. They have a direct impact on hospital resource utilization and patient health, and may even lead to patient dissatisfaction. Reducing health inequalities requires addressing the factors that contribute to low patient engagement in healthcare. Individuals who consistently miss appointments may have significant unmet medical needs; this represents an understudied population. Thus, missed general practitioner appointments at the individual level may serve as a risk indicator for vulnerability and adverse health outcomes.

Alawadhi A et al. evaluated the rate and predictors of missed hospital appointments, as well as variability in contributing factors, across multiple outpatient clinics at the Royal Hospital, Sultanate of Oman, between 2014 and 2018 [12]. During this period, there were 769,118 scheduled outpatient appointments. The overall rate of missed hospital appointments was 22.3% (n = 171,889), varying between clinics (14.0% for Oncology and 30.3% for Urology). Important predictors included age, sex, service costs, distance of the patient’s residence from the hospital, waiting time, and appointment day and season. Predictors of missed appointments varied in their effects across clinics. The authors concluded that interventions to reduce missed appointment rates should take these factors into account and be tailored to the specific clinic.

Alawadhi A et al. also conducted a survey among a randomly selected sample of patients who missed their outpatient appointments at the Royal Hospital, Sultanate of Oman, from March to April 2021, across six clinics [13]. Patients were interviewed via telephone using a structured survey, while a self-administered survey was distributed to medical staff to explore their perspectives. A total of 288 patients and 52 medical staff members participated. Patients most frequently cited lack of transportation (11.4%), ceasing to require consultation (7.5%), and forgetting the appointment (6.8%) as reasons for missing appointments. Staff members most commonly identified transportation issues (23.8%), not receiving an SMS (16.9%), and forgetting the appointment (15.4%) as the main reasons. The frequency of these causes differed significantly among clinics. In the obstetrics clinic (OR 9.48; 95% CI 2.66-33.78) and the diabetes clinic (OR 10.55; 95% CI 2.68-38.58), family responsibilities were the primary theme. In the oncology clinic (OR 4.83; 95% CI 1.11-21.02), transportation was the key issue. Suggested improvements primarily focused on enhancing the telephone reminder system, introducing flexible appointment scheduling, and refining the overall reminder system.

Anisi S et al. conducted a cross-sectional study that included all outpatients with scheduled appointments between March 20, 2016, and March 20, 2017 (n = 148,077) [14]. Of these, 50.1% of patients did not show up. The highest and lowest no-show rates were observed in the general practice (80.3%) and nephrology (40.1%) clinics, respectively. Appointment lead times averaged 10.2 (±14.7) days overall, while no-show and attending patient lead times averaged 11.7 (±15.6) and 8.8 (±13.7) days, respectively (P < 0.001). Variables found to predict patient no-shows included lead times exceeding two weeks (OR = 1.80), web-based appointment systems (OR = 1.71), interactive voice response systems (OR = 1.69), month of appointment (OR = 1.03), and clinic working shift (OR = 0.94).

Giunta DH et al. conducted a retrospective cohort study of all adult outpatients aged over 18 years who requested at least one scheduled ambulatory medical appointment between January 1, 2015 and December 31, 2016 at a university hospital in Buenos Aires, Argentina [15]. The overall nonattendance rate was 27.84% (95% CI, 27.79-27.90). The nonattendance rate for general practitioner appointments was 25.53% (95% CI, 25.42-25.63), for clinical specialties 27.78% (95% CI, 27.68-27.87), and for surgical specialties 29.31% (95% CI, 29.23-29.40). Based on the analysis of the results, the authors concluded that local estimates should be used in designing effective interventions to improve adherence to scheduled outpatient healthcare appointments.

Magadzire BP et al. reviewed medical records from 2014 to 2015 to determine whether missed appointments for collecting medicine parcels were indicative of loss to follow-up and to identify patient- and system-level factors associated with missed appointments [16]. There was a 33% no-show rate during this period. The reasons included temporary shifts to private care, relocation, and forgetting work commitments or appointments. While most healthcare professionals acknowledged these barriers to attendance, some believed they were outside their professional scope. Furthermore, clinicians noted that underutilization of medications, lack of patient responsibility, and the use of multiple healthcare providers (such as traditional healers) were contributing factors to missed appointments.

In another study, Giunta D et al. conducted a cohort study of adult patients who had a scheduled outpatient appointment for primary care between January 2010 and July 2011 at the Italian Hospital of Buenos Aires [17]. Of the 113,716 appointments included, 25,687 (22.7%; 95% CI, 22.34-22.83%) were not attended. Age (OR 0.99; 95% CI, 0.99-0.99), number of issues in the personal health record (OR 0.98; 95% CI, 0.98-0.99), time interval between appointment request and appointment date (OR 1.00; 95% CI, 1.00-1.00), history of nonattendance (OR 1.07; 95% CI, 1.07-1.07), appointments after 4 p.m. (OR 1.30; 95% CI, 1.24-1.35), and particular days of the week (OR 1.00; 95% CI, 1.06-1.10) were all statistically significantly associated with nonattendance.

In a nationwide retrospective cohort analysis, Ellis DA et al. extracted general practice data from the UK National Health Service routinely collected across Scotland between September 5, 2013 and September 5, 2016 [18]. Using a negative binomial model offset by the number of appointments made, the authors calculated the per-patient number of missed appointments and examined the likelihood of missing a general practice appointment. They evaluated the impact of practice-level factors (e.g., appointment availability and geographic location) and patient-level variables (e.g., age, sex, and socioeconomic status) on the likelihood of nonattendance. Out of 909,073 patients with scheduled appointments, 550,083 were included in the analysis after data processing. During the three-year study period, 104,461 (19.0%) patients missed more than two appointments. Distinct patterns of nonattendance emerged after adjusting for the total number of appointments. Patients with low socioeconomic status and those aged 16-30 years (RR 1.21; 95% CI, 1.19-1.23) or over 90 years (RR 2.20; 95% CI, 2.09-2.29) were significantly more likely to miss multiple appointments. Although men were slightly more likely to miss appointments in the adjusted model, women missed a higher overall number of appointments. The authors concluded that both patient- and practice-related factors contribute to general practice appointment nonattendance.

The evidence suggests that all patients should receive a reminder, or “reminder plus,” three days in advance unless they specifically request otherwise. This is because such reminders actively encourage patients who are unable to attend to cancel their appointments and reschedule if needed. Since the timing of a reminder, between one and seven days before the scheduled appointment, has no significant effect on patients’ attendance habits, three days is considered sufficient time to either accommodate a patient’s cancellation, allow the healthcare service to reallocate the appointment to another patient, or provide the clinician time to complete administrative tasks related to patient care [19,20].

The usefulness of reminder systems for encouraging attendance, cancellations, and rescheduling of appointments across healthcare settings, as well as among specific patient groups, and the contextual factors indicating that reminders are underutilized, were investigated in a systematic review by McLean SM et al. [21,22]. They identified six areas of inefficiency, suggesting that reminder systems are not being used to their full potential. Every patient should receive a reminder to assist in attending their medical appointments unless otherwise specified. The type of reminder system selected should be tailored to the needs of the specific service. The authors concluded that supportive administrative procedures are essential for healthcare services to improve attendance, cancellations, rescheduling, and redistribution of appointments to other patients, thereby maximizing the efficiency of appointment and reminder systems.

There are several limitations to this study. The present study included responses and analysis from a single hospital in the North Sharqiya Governorate. We acknowledge that the reasons for missed appointments may differ across other governorates; therefore, these findings cannot be generalized to represent the entire country. Another limitation is that the reasons were determined based on phone interviews without in-depth analysis of social and personal factors that may have directly or indirectly contributed to defaulting. As the study relied solely on telephone responses, response bias is possible. The study gathered responses and analyzed data from all available departments in the hospital. However, we recognize that patients’ priority in attending appointments for certain departments may depend on the indication (e.g., routine follow-up or an underlying health concern), which could influence the seriousness with which they attend scheduled appointments.

## Conclusions

A number of factors, including patient forgetfulness, work-related constraints, and transportation difficulties, contribute to missed outpatient appointments at Ibra Hospital. Many patients also bypass official appointment scheduling systems, highlighting the need for administrative process simplification. To reduce no-show rates, it is essential to implement efficient reminder systems and provide flexible scheduling options. Additionally, addressing logistical barriers such as transportation can further improve patient adherence. These findings underscore the necessity of focused, context-specific interventions to enhance appointment attendance and overall healthcare delivery outcomes.
